# Left Atrial Hypertension, Electrical Conduction Slowing, and Mechanical Dysfunction – The Pathophysiological Triad in Atrial Fibrillation-Associated Atrial Cardiomyopathy

**DOI:** 10.3389/fphys.2021.670527

**Published:** 2021-08-05

**Authors:** Martin Eichenlaub, Bjoern Mueller-Edenborn, Jan Minners, Nikolaus Jander, Martin Allgeier, Heiko Lehrmann, Simon Schoechlin, Juergen Allgeier, Dietmar Trenk, Franz-Josef Neumann, Thomas Arentz, Amir Jadidi

**Affiliations:** Division of Cardiology and Angiology II, University Heart Center Freiburg-Bad Krozingen, Bad Krozingen, Germany

**Keywords:** atrial fibrillation, pathophysiology, left atrial strain, pulmonary vein isolation, left atrial pressure, atrial cardiomyopathy, atrial conduction abnormalities

## Abstract

**Background:**

Atrial fibrillation (AF) is the most common arrhythmia and a significant burden for healthcare systems worldwide. Presence of relevant atrial cardiomyopathy (ACM) is related to persistent AF and increased arrhythmia recurrence rates after pulmonary vein isolation (PVI).

**Objective:**

To investigate the association of left atrial pressure (LAP), left atrial electrical [invasive atrial activation time (IAAT) and amplified p-wave duration (aPWD)] and mechanical [left atrial emptying fraction (LA-EF) and left atrial strain (LAS)] functional parameters with the extent of ACM and their impact on arrhythmia recurrence following PVI.

**Materials and Methods:**

Fifty patients [age 67 (IQR: 61–75) years, 78% male] undergoing their first PVI for persistent AF were prospectively included. LAP (maximum amplitude of the v-wave), digital 12-lead electrocardiogram, echocardiography and high-density endocardial contact mapping were acquired in sinus rhythm prior to PVI. Arrhythmia recurrence was assessed using 72-hour Holter electrocardiogram at 6 and 12 months post PVI.

**Results:**

Relevant ACM (defined as left atrial low-voltage extent ≥2 cm^2^ at <0.5 mV threshold) was diagnosed in 25/50 (50%) patients. Compared to patients without ACM, patients with ACM had higher LAP [17.6 (10.6–19.5) mmHg with ACM versus 11.3 (7.9–14.0) mmHg without ACM (*p* = 0.009)]. The corresponding values for the electrical parameters were 166 (149–181) ms versus 139 (131–143) ms for IAAT (*p* < 0.0001), 163 (154–176) ms versus 148 (136–152) ms for aPWD on surface-ECG (*p* < 0.0001) and for the mechanical parameters 27.0 (17.5–37.0) % versus 41.0 (35.0–45.0) % for LA-EF in standard 2D-echocardiography (*p* < 0.0001) and 15.2 (11.0–21.2) % versus 29.4 (24.9–36.6) % for LAS during reservoir phase (*p* < 0.0001). Furthermore, all parameters showed a linear correlation with ACM extent (*p* < 0.05 for all). Receiver-operator-curve-analysis demonstrated a LAP ≥12.4 mmHg [area under the curve (AUC): 0.717, sensitivity: 72%, and specificity: 60%], a prolonged IAAT ≥143 ms (AUC: 0.899, sensitivity: 84%, and specificity: 80%), a prolonged aPWD ≥153 ms (AUC: 0.860, sensitivity: 80%, and specificity: 79%), an impaired LA-EF ≤33% (AUC: 0.869, sensitivity: 84%, and specificity: 72%), and an impaired LAS during reservoir phase ≤23% (AUC: 0.884, sensitivity: 84%, and specificity: 84%) as predictors for relevant ACM. Arrhythmia recurrence within 12 months post PVI was significantly increased in patients with relevant ACM ≥2 cm^2^, electrical dysfunction with prolonged IAAT ≥143 ms and mechanical dysfunction with impaired LA-EF ≤33% (66 versus 20, 50 versus 23 and 55 versus 25%, all *p* < 0.05).

**Conclusion:**

Left atrial hypertension, electrical conduction slowing and mechanical dysfunction are associated with ACM. These findings improve the understanding of ACM pathophysiology and may be suitable for risk stratification for new-onset AF, arrhythmia recurrence following PVI, and development of novel therapeutic strategies to prevent AF and its associated complications.

## Introduction

Atrial fibrillation (AF) is the most common arrhythmia worldwide and associated with significant morbidity and mortality (Hindricks et al., 2020). Atrial cardiomyopathy (ACM) is characterized by progressive fibrosis of healthy atrial myocardium and is present in the majority of patients with persistent forms of AF (Platonov et al., 2011; Haissaguerre et al., 2016). ACM is related to adverse outcomes in AF, including higher arrhythmia recurrence rates after pulmonary vein isolation (PVI) (Verma et al., 2005; Marrouche et al., 2014).

The development of ACM itself, and the consequences to AF-persistency, were investigated in a variety of clinical studies. ACM was found to impair left atrial function on several levels: First, ACM was associated with impaired left atrial mechanical function as evidenced by standard 2D-echocardiography and speckle tracking (Pilichowska-Paszkiet et al., 2018; Seewoster et al., 2019). Second, ACM affects the electrical function of the left atrium, as is evidenced by progressive intra-atrial conduction slowing in these patients (Jadidi et al., 2018; Muller-Edenborn et al., 2020). Third, ACM was found to be associated with elevated left atrial pressures, likely as a result of decreased atrial compliance in a vicious circle promoting further adverse fibrotic atrial remodeling (Park et al., 2014; Roh et al., 2020). However, all this information is mostly derived from individual studies investigating one single aspect of this pathophysiological concept. The aim of the current study, in contrast, was to perform a holistic determination of all these aspects in a single patient cohort, in order to understand their relative expression and importance in ACM and AF.

## Materials and Methods

We prospectively included 50 consecutive patients with persistent AF undergoing their first PVI. All patients underwent electrical cardioversion 4–6 weeks prior to PVI. Digital 12-lead electrocardiogram (ECG) and transthoracic echocardiography (TTE) were performed in sinus rhythm 1 day prior to PVI. The following day, after completion of the transseptal access to the left atrium, we invasively measured left atrial pressure (LAP) and subsequently performed a high-density endocardial voltage and activation mapping as well as PVI in sinus rhythm. Follow-up visits were assessed 6 and 12 months after PVI to monitor arrhythmia recurrence. The study was approved by the Institutional Review Board and all patients provided written informed consent prior to enrolment.

### Digital 12-Lead-ECG

We recorded standard digital 12-lead ECG in all patients during sinus rhythm 1 day prior to PVI using a AT-104 PC (Schiller, Baar, Switzerland). Duration of the amplified p-wave (aPWD; 40 mm/mV amplification and 200 mm/s sweep speed) was measured between the earliest and latest component of the p-wave in any of the 12 electrocardiographic leads as described in previous studies (Jadidi et al., 2018; Muller-Edenborn et al., 2020).

### Transthoracic Echocardiography

We performed standardized 2D TTE (GE ultrasound system E95, M5Sc probe, GE Healthcare, Solingen, Germany) in all patients during sinus rhythm 1 day prior to PVI. Left atrial diameter, left atrial volume index, left ventricular end-diastolic dimension (LVEDD) and left ventricular ejection fraction (LV-EF) were measured in accordance with current guidelines (Lang et al., 2015). Left atrial mechanical function was analyzed by measurement of biplane left atrial emptying fraction (LA-EF) and biplane 2D speckle tracking in four- and two-chamber views (frame rate between 57 and 90 frames per second) (Badano et al., 2018; Walek et al., 2020): LA-EF was calculated with the following formula: (LA maximum volume − LA minimum volume) / LA maximum volume × 100%. We analyzed left atrial global longitudinal strain (left atrial strain, LAS) using TomTec software (AutoStrain, TomTec Imaging Systems, Unterschleissheim, Germany). Thereby, a complete RR-cycle (end-diastole to end-diastole) was automatically selected and endocardial borders were automatically placed. Subsequently, LAS was measured automatically in the reservoir phase (LASr, between mitral valve closure and mitral valve opening), conduit phase (LAScd, between mitral valve opening and onset of LA contraction), and contraction phase (LASct, between onset of LA contraction and mitral valve closure). All automatically performed measurements were rechecked by a physician who intervened in the case of relevant deviations.

### Measurement of Left Atrial Pressure, Endocardial Contact Mapping and Ablation Procedure

Pulmonary vein isolation was performed under general anesthesia. A long sheath (Swartz^TM^ braided transseptal guiding introducer Lamp 45^TM^, Abbott, United States) was used for transseptal puncture. Immediately after transseptal puncture, LAP was recorded through the transseptal needle (BRK-1^TM^ transseptal needle, Abbott, United States). The maximum amplitude of the v-wave was measured when stable sinus rhythm was present with a rate between 60 and 80 bpm. Subsequently, we acquired high-density endocardial activation and voltage maps (mean 3052 ± 1225 sites) in sinus rhythm using either a 20-polar Lasso-Nav (variable diameter: 15–25 mm, Biosense Webster, Irvine, CA, United States) or a PentaRay-Nav catheter (electrode size for both catheters: 1 mm, spacing: 2-6-2 mm, Biosense Webster, Irvine, CA, United States) in combination with the electro-anatomical contact mapping system CARTO-3 (Biosense Webster, Irvine, CA, United States) as described previously (Jadidi et al., 2016, 2018). Invasive atrial activation time (IAAT) was measured between the beginning of the amplified p-wave on surface ECG and the latest activated site in the left atrium. Extent of ACM was measured as the area of bipolar left atrial low-voltage substrate (LA-LVS) <0.5 mV. Relevant ACM was defined as a LA-LVS extent ≥2 cm^2^ as prespecified in accordance with prior studies (Jadidi et al., 2018; Muller-Edenborn et al., 2020).

After completion of left atrial mapping, proximal circumferential PVI was performed using an irrigated-tip contact force-enabled radiofrequency ablation catheter (Smart Touch Thermocool, tip electrode: 3.5 mm, spacing: 2-5-2 mm, Biosense Webster, Irvine, CA, United States). An ablation index of 350–380 at the posterior left atrium (at 30 W) and of 450–500 at other areas (at 35 W) and an inter-lesion distance <5 mm were aimed. Achievement of bi-directional entrance and exit block was defined as acute procedural success.

### Follow-Up

Ambulatory clinical visits including 12-lead ECG and 72-hour Holter ECG 6 and 12 months after PVI were scheduled for all patients. Arrhythmia recurrence was evaluated in absence of antiarrhythmic therapy and was defined as any documented episode of AF, atypical atrial flutter or atrial tachycardia lasting >30 s after a 3-month blanking period. In symptomatic patients in whom no arrhythmia recurrence could be recorded, the patients received an event recorder allowing to record a single lead ECG during symptomatic episodes.

### Endpoints

The primary endpoint was to investigate the relation of ACM with LAP, mechanical and electrical parameters.

The secondary endpoint was to assess the predictive value of these parameters regarding arrhythmia recurrence at 12 months following PVI.

### Statistical Analysis

SPSS Statistics 23 (IBM, New York, NY, United States) and GraphPad Prism 8 (GraphPad Software, San Diego, CA, United States) were used for statistical analysis. We used Shapiro–Wilk test to assess test for normality. Normally distributed data are given as mean ± SD and skewed distributed data as median with interquartile range (IQR, 1st and 3rd quartiles). For group comparison, Student’s *t*-test and Mann–Whitney *U* test was performed depending on the number of groups and distribution. We used Fisher’s exact test to compare categorical variables. Cut-off values for ACM diagnosis using left atrial pressure, mechanical and electrical parameters were acquired using receiver-operating curves (ROC). Univariate linear regression analysis was conducted for all independent variables. All univariate variables with *p* < 0.05 were selected for analysis in the multivariate model. We used Kaplan–Meier curves to illustrate arrhythmia recurrence and compared them using the log-rank test. Cox proportional hazard regression models were used to analyze the impact of clinical covariates on arrhythmia recurrence. A two-tailed *p* < 0.05 was considered significant.

## Results

Patient characteristics and procedural data are illustrated in Table 1. Patients were on average 67 (IQR: 61–75) years old and predominantly male (78%). Relevant ACM was diagnosed in 25/50 (50%) patients. In the entire study cohort median LA-LVS extent at <0.5 mV threshold was 2.5 (IQR: 0.4–17.9) cm^2^. The median LA-LVS extent in patients without versus those with ACM was 0.4 (0.0–1.1) cm^2^ versus 17.7 (4.6–29.1) cm^2^. No significant differences were observed in patients with and without ACM with regard to conventional cardiovascular risk factors such as atrial hypertension, diabetes mellitus or coronary artery disease. Left-ventricular dysfunction or valvulopathies were present in only a minority of patients in both groups.

**TABLE 1:** Clinical and procedural characteristics.

	All patients (*n* = 50)	No ACM (*n* = 25)	ACM (*n* = 25)	*p*-Value
Age, years	67 (61–75)	61 (53–72)	71 (65–77)	0.002
Male sex, *n* (%)	39 (78)	22 (88)	17 (68)	0.171
BMI, kg/m^2^	28 ± 4	29 ± 4	26 ± 4	0.010
Hypertension, *n* (%)	33 (66)	14 (56)	19 (76)	0.232
Diabetes mellitus, *n* (%)	4 (8)	1 (4)	3 (12)	0.609
Prior stroke or TIA, *n* (%)	1 (2)	0 (0)	1 (4)	1.0
Structural cardiomyopathy, *n* (%)	8 (16)	3 (12)	5 (20)	0.702
Coronary artery disease, *n* (%)	7 (14)	2 (8)	5 (20)	0.417
CHA_2_DS_2_-VASc-Score	3 (1–3)	2 (0–3)	3 (2–4)	0.004
Prior antiarrhythmic therapy, *n* (%)	41 (82)	20 (80)	21 (84)	1.0
Antiarrhythmic therapy on admission day, *n* (%)	31 (62)	16 (64)	15 (60)	1.0
Amiodarone, *n* (%)	19 (38)	7 (28)	12 (48)	0.244
Flecainide, *n* (%)	5 (10)	4 (16)	1 (4)	0.349
Sotalol, *n* (%)	5 (10)	3 (12)	2 (8)	1.0
Dronedarone, *n* (%)	1 (2)	1 (4)	0 (0)	1.0
Propafenone, *n* (%)	1 (2)	1 (4)	0 (0)	1.0
LA diameter, mm	46 ± 6	44 ± 5	47 ± 6	0.066
LA volume index, mL/m^2^	49 ± 14	46 ± 12	53 ± 14	0.082
LVEF, %	57 ± 8	57 ± 8	56 ± 8	0.877
LV dysfunction with LVEF <50%, *n* (%)	11 (22)	6 (24)	5 (20)	1.0
LVEDD, mm	54 ± 5	54 ± 5	53 ± 6	0.648
LV cavity dilatation, *n* (%)	9 (18)	2 (8)	7 (28)	0.138
Relevant (at least moderate) mitral valve regurgitation, *n* (%)	3 (6)	1 (4)	2 (8)	1.0
LA-LVS extent, cm^2^	2.5 (0.4–17.9)	0.4 (0.0–1.1)	17.7 (4.6–29.1)	<0.001
Procedure duration*, min	144 ± 23	140 ± 24	148 ± 21	0.19
Successful circumferential PVI, *n* (%)	50 (100)	25 (100)	25 (100)	1.0
Additional CTI ablations, *n* (%)	8 (16)	5 (20)	3 (12)	0.70
Fluoroscopy time for EPS and AF ablation procedure, min	5.4 ± 3.2	5.1 ± 3.2	5.6 ± 3.3	0.56
Effective dose during EPS and AF ablation procedure, mSv	0.09 (0.05–0.16)	0.09 (0.04–0.21)	0.09 (0.05–0.15)	0.82

ACM, atrial cardiomyopathy; AF, atrial fibrillation; BMI, body mass index; CTI, cavotricuspid isthmus; EDD, end-diastolic diameter; EF, ejection fraction; EPS, electrophysiological study; LA, left atrial; LV, left ventricular; LVS, low-voltage substrate; PVI, pulmonary vein isolation; TIA, transient ischemic attack.

*Time between start of the first venous puncture until removal of the catheters.

### Pathophysiological Alterations in Atrial Cardiomyopathy

**FIGURE 1:**
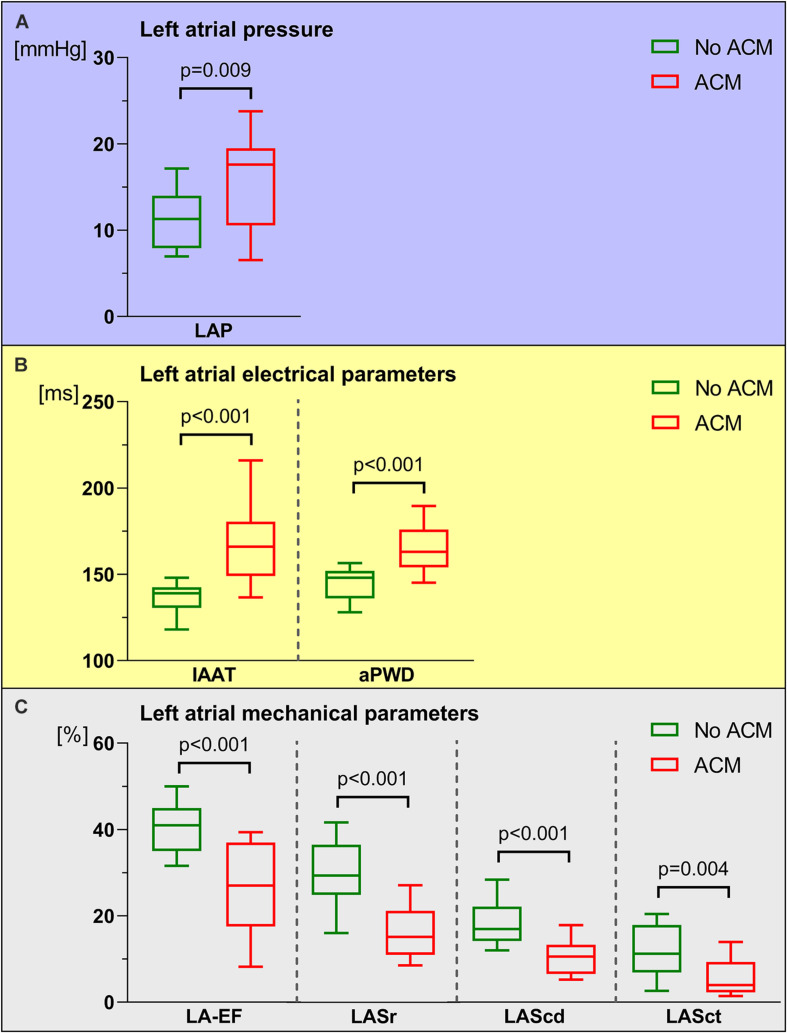
Comparison between left atrial pressure (LAP), left atrial electrical and left atrial mechanical parameters in patients with and without atrial cardiomyopathy (ACM). In patients with ACM (red boxes and whiskers), LAP **(A)** and left atrial electrical parameters [invasive atrial activation time (IAAT), amplified p-wave duration (aPWD), **(B)**] were significantly increased. In contrast, left atrial mechanical parameters [left atrial emptying fraction (LA-EF), left atrial strain during reservoir phase (LASr), conduit phase (LAScd), and contraction phase (LASct), **(C)** were significantly reduced in ACM compared to patients without ACM (green boxes and whiskers). Boxes include data between lower and upper quartiles and whiskers mark 10th and 90th percentiles.

**FIGURE 2:**
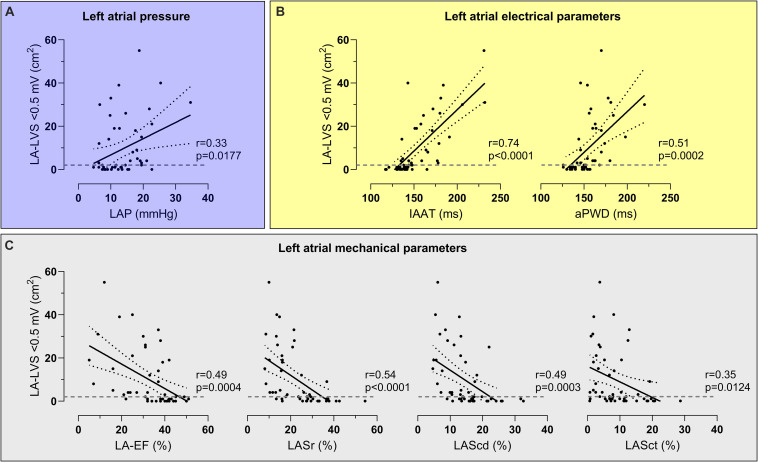
Correlations between atrial cardiomyopathy (ACM) extent and left atrial pressure (LAP), electrical parameters and mechanical parameters. LAP **(A)**, left atrial electrical parameters [invasive atrial activation time (IAAT), amplified p-wave duration (aPWD), **(B)**] and left atrial mechanical parameters [left atrial emptying fraction (LA-EF), left atrial strain during reservoir phase (LASr), conduit phase (LAScd), and contraction phase (LASct), **(C)**] correlated significantly with ACM extent [assessed as left atrial area displaying low-voltage substrate (LA-LVS) <0.5 mV]. The dashed line marks the border between absence (<2 cm^2^ LA-LVS extent at 0.5 mV threshold) and presence of relevant ACM.

Patients with ACM demonstrated characteristic alterations in key left atrial functions: Compared to patients without ACM, patients with ACM had on average higher LAP [17.6 (10.6–19.5) mmHg with ACM versus 11.3 (7.9–14.0) mmHg] without ACM (*p* = 0.009; Figure 1A). Patients with ACM were also subject to significant electrical conduction slowing both evidenced using invasively measured IAAT [166 (149–181) ms versus 139 (131–143) ms, *p* < 0.0001] and aPWD from the surface-ECG [163 (154–176) ms versus 148 (136–152) ms, *p* < 0.0001; Figure 1B]. Impaired LA mechanical function in ACM was evident both by standard 2D-echocardiography [LA-EF: 27.0 (17.5–37.0) % in ACM versus 41.0 (35.0–45.0) % without ACM, *p* < 0.0001], as well as by speckle tracking-imaging of the individual phases of the left atrial cardiac cycle [15.2 (11.0–21.2) % versus 29.4 (24.9–36.6) % for LASr, *p* < 0.0001; 10.6 (6.5–13.3) % versus 17.0 (14.2–22.2) % for LAScd, *p* < 0.0001; and 4.0 (2.3–9.3) % versus 11.2 (6.9–17.9) % for LASct, *p* = 0.004; Figure 1C]. Correlations between ACM extent and LAP, electrical as well as mechanical parameters are shown in Figure 2. None of the automatically performed LAS measurements showed relevant deviations and therefore no manual adjustment was done in order to keep the results investigator-independent and universally valid.

### Determination of Pathological Cut-Off Values and Outcome-Validation

**FIGURE 3:**
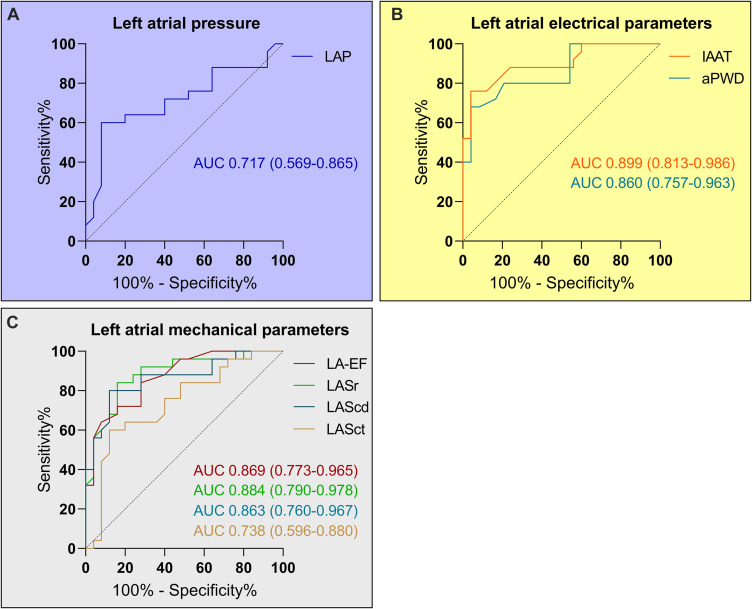
Diagnosis of relevant atrial cardiomyopathy (ACM) based on left atrial pressure (LAP), left atrial electrical and mechanical parameters. Receiver-operating curve analysis determined a LAP ≥12.4 mmHg as predictor for relevant ACM with a sensitivity of 72% and a specificity of 60% **(A)**. The corresponding values for the left atrial electrical parameters **(B)** were ≥143 ms (sensitivity 84% and specificity 80%) for the invasive atrial activation time (IAAT) and ≥153 ms (sensitivity 80% and specificity 79%) for the amplified p-wave duration (aPWD). The values for the left atrial mechanical parameters **(C)** were ≤33% for LA-EF (sensitivity 84% and specificity 72%), ≤23% for LASr (sensitivity 84% and specificity 84%), ≤13.4% for LAScd (sensitivity 88% and specificity 80%), and ≤6.8% for LASct (sensitivity 80% and specificity 64%).

Receiver-operator-curve-analysis demonstrated at least moderate diagnostic abilities for all investigated parameters (Figures 3A–C). The best classification was made using either electrical parameters (c-statistic of 0.899 and 0.860 for IAAT and aPWD, respectively; Figure 3B) or speckle-tracking echocardiography (c-statistic of 0.884 for LASr; Figure 3C). Using the Youden-index, the optimal cut-off values to predict ACM were LAP ≥12.4 mmHg, IAAT ≥143 ms, aPWD ≥153 ms, LA-EF ≤33%, LASr ≤23%, LAScd ≤13.4%, and LASct ≤6.8%.

**FIGURE 4:**
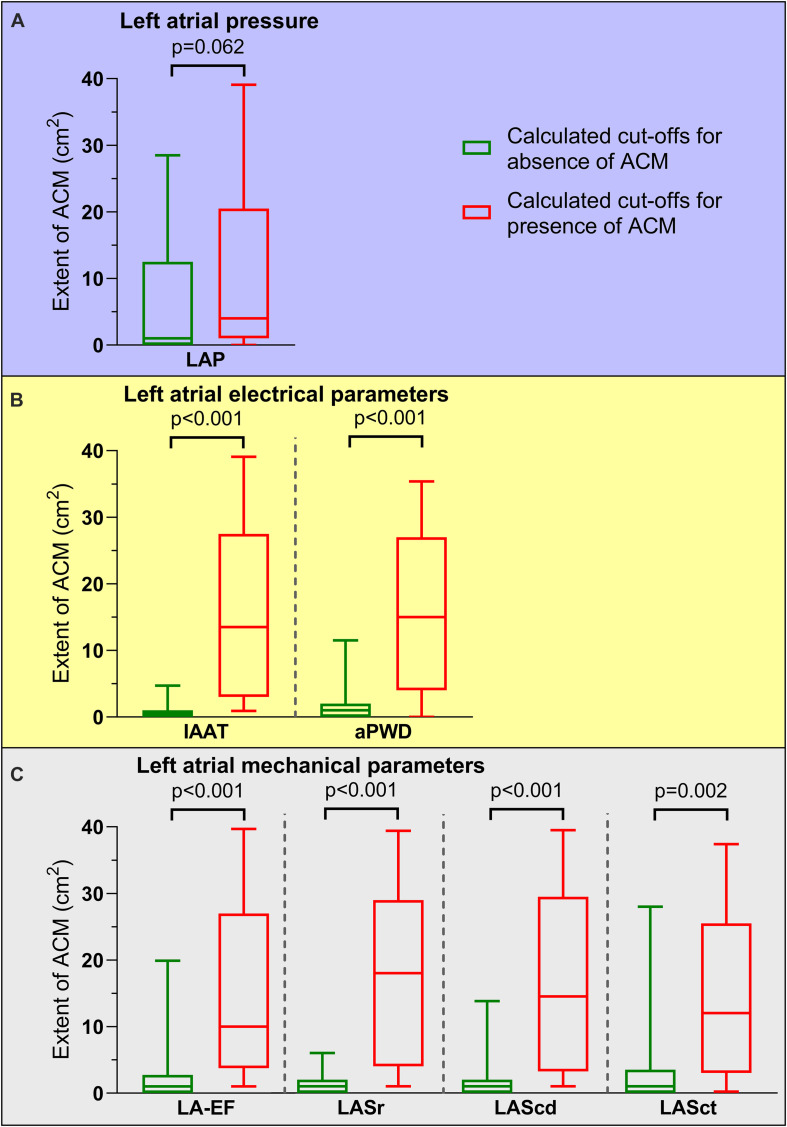
Association of pathological cut-off values for left atrial function and atrial cardiomyopathy (ACM) extent. ACM extent for patients with calculated cut-off values for presence of ACM (red boxes and whiskers) compared to cut-off values for absence of ACM (green boxes and whiskers) are depicted. With the exception of left atrial pressure [LAP, **(A)**], all left atrial electrical [invasive atrial activation time (IAAT), amplified p-wave duration (aPWD), **(B)**] and left atrial mechanical parameters [left atrial emptying fraction (LA-EF), left atrial strain during reservoir phase (LASr), conduit phase (LAScd), and contraction phase (LASct), **(C)**] allowed significant differentiation between high and low ACM extent: 4.3 (0.7–20.6) cm^2^ versus 1.1 (0.2–12.2) cm^2^ for LAP, 13.1 (3.2–27.6) cm^2^ versus 0.5 (0–1.2) cm^2^ for IAAT, 14.7 (4.0–26.9) cm^2^ versus 0.8 (0.2–1.8) cm^2^ for aPWD, 9.6 (3.8–26.6) cm^2^ versus 0.7 (0–2.8) cm^2^ for LA-EF, 17.7 (4.1–29.1) cm^2^ versus 0.5 (0.1–1.5) cm^2^ for LASr, 14.6 (3.5–29.4) cm^2^ versus 0.6 (0.1–1.9) cm^2^ for LAScd, and 11.5 (3.0–25.0) cm^2^ versus 0.7 (0.2–3.7) cm^2^ for LASct. Boxes include data between lower and upper quartiles and whiskers mark 10th and 90th percentiles.

With the exception of LAP, application of these calculated cut-offs allowed to differentiate accurately between patients with and without relevant ACM extent (Figures 4A–C).

In univariate analysis, LAP, electrical parameters and mechanical parameters together with age, BMI and CHA_2_DS_2_-VASc-Score, were associated with ACM (Table 2). In multivariate analysis, LAP, electrical parameters and mechanical parameters remained as significant predictors for relevant ACM (Table 2).

**TABLE 2:** Predictors for atrial cardiomyopathy.

	Univariate regression analysis		Multivariate regression analysis (adjusted to age, BMI, CHA_2_DS_2_-VASc-Score)	
	Odds ratio (95% CI)	*p*-Value	Odds ratio (95% CI)	*p*-Value
LAP ≥12.4 mmHg	3.86 (1.18–12.61)	0.025	5.24 (1.14–24.15)	0.034
IAAT ≥143 ms	23.22 (5.10–105.73)	<0.001	20.37 (3.09–134.36)	0.002
aPWD ≥153 ms	15.20 (3.79–70.00)	<0.001	39.65 (4.24–371.28)	0.001
LA-EF ≤33%	13.50 (3.40–53.68)	<0.001	35.06 (3.79–324.01)	0.002
LASr ≤23%	27.56 (6.08–125.04)	<0.001	37.41 (4.81–290.87)	0.001
LAScd ≤13.4%	21.00 (4.92–89.56)	<0.001	16.92 (3.22–89.00)	0.001
LASct ≤6.8%	7.11 (1.99–25.47)	0.003	44.06 (3.73–520.64)	0.003
Age, years	1.13 (1.04–1.22)	0.003		
BMI, kg/m^2^	0.81 (0.69–0.96)	0.016		
CHA_2_DS_2_-VASc-Score	1.79 (1.18–2.73)	0.007		

*aPWD, amplified p-wave duration; BMI, body mass index; cd, conduit phase; ct, contraction phase; LA-EF, left atrial emptying fraction; IAAT, invasive atrial activation time; LAP, left atrial pressure; LAS, left atrial strain; r, reservoir phase.*

**FIGURE 5:**
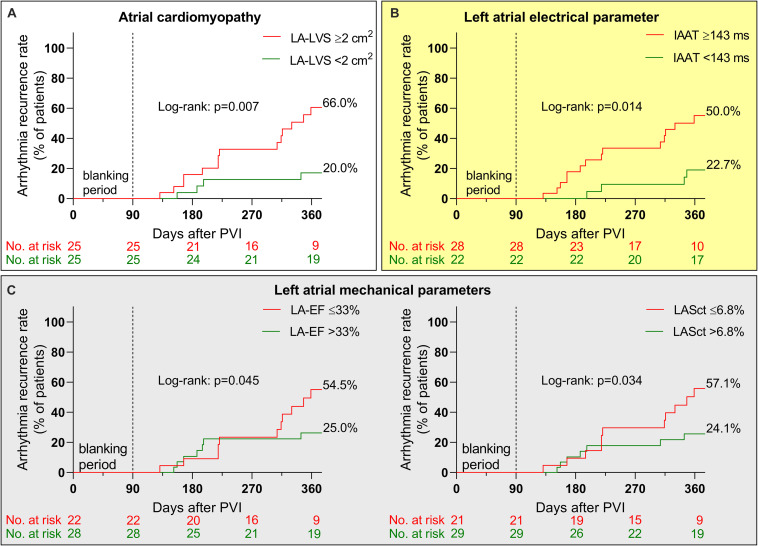
Arrhythmia recurrence after pulmonary vein isolation (PVI). Kaplan–Meier curves illustrate arrhythmia recurrence after PVI in patients with relevant atrial cardiomyopathy [ACM defined as ≥2 cm^2^ left atrial low-voltage substrate (LA-LVS) extent at 0.5 mV threshold, red curve in **A**], prolonged left atrial electrical conduction parameters [invasive atrial activation time (IAAT), red curve in **B**] and impaired left atrial mechanical parameters [left atrial emptying fraction (LA-EF) and left atrial strain during contraction phase (LASct), red curves in **C**] compared to patients with normal cut-offs (green curves).

Nineteen out of fifty patients (38%) experienced arrhythmia recurrence within 12 months following PVI. Relevant ACM as defined by endocardial contact mapping was related to arrhythmia recurrence [HR 3.79 (95% CI 1.4–10.6); Figure 5A], as were IAAT [HR 3.38 (95% CI 1.2–9.5); Figure 5B], LA-EF [HR 2.55 (95% CI 1.0–6.6); Figure 5C) and LASct [HR 2.64 (95% CI 1.0–6.8); Figure 5C]. LAP, aPWD, LASr and LAScd did not reach significance (Supplementary Figure 1).

## Discussion

The current study reports three main findings that advance our understanding of ACM in the context of AF. First, ACM is a multifactorial disease that affects left atrial pressure, left atrial electrical and mechanical function. Second and conversely, ACM can be diagnosed with reasonable diagnostic validity using defined cut-off values for any of the above mentioned aspects of ACM with the exception of LAP. Third, diagnosis of ACM is clinically relevant as it relates to arrhythmia freedom following PVI.

### Definition of Atrial Cardiomyopathy

The current EHRA/HRS/APHRS/SOLAECE consensus statement defines ACM as any complex of structural, architectural, contractile or electrophysiological changes affecting the atria with the potential to produce clinically relevant manifestations (Goette et al., 2017). According to histological/pathophysiological changes four EHRAS-classes were defined: class I: principal cardiomyocyte changes, class II: principally fibrotic changes, class III: combined cardiomyocyte-pathology/fibrosis, and class IV: primarily non-collagen infiltration (Goette et al., 2017). The fibrotic remodeling of the atria is typically found in patients with AF. Moreover, the persistency of AF and clinical AF type are predictors for increased fibrotic remodeling (persistent AF is more affected by fibrosis than paroxysmal AF). In contrast, in patients without history of AF, fibrotic atrial remodeling is absent (Platonov et al., 2011).

### Imaging and Mapping Methods for Atrial Cardiomyopathy

In the current study, endocardial contact mapping was used to quantify ACM. The definition of LA-LVS is still debated due to the lack of histological examination and the dependence of atrial electrogram voltages on the underlying rhythm including both the wave front direction and the cycle length (Wong et al., 2019). Moreover, the size of mapping electrodes and inter-electrode spacing as well as the electrode-tissue contact influence the recorded voltage amplitudes (Anter et al., 2015). Sanders et al. (2003) defined a bipolar voltage amplitude ≤0.05 mV as scar and an amplitude ≤0.5 mV as low-voltage substrate in sinus rhythm (Sanders et al., 2003). Subsequently, the cut-off of ≤0.5 mV was used to define LA-LVS in many other studies confirmed by the fact that these areas incorporate most arrhythmogenic slow conduction substrate related to AF and atrial tachycardia development (Verma et al., 2005; Rolf et al., 2014; Yamaguchi et al., 2014; Jadidi et al., 2016, 2020). A recent study revealed that bipolar low-voltage areas <0.5 mV as defined by endocardial high-density mapping with small electrodes (1 mm electrodes with 2-6-2 mm spacing) correspond very well to unipolar LA-LVS. Therefore, previously reported dependency of voltage amplitudes to wavefront-to-bipole direction is not of high clinical importance in both sinus rhythm and AF due to the high number of bipolar voltage recordings with different bipole orientations when using high-density (multi-electrode) mapping (Nairn et al., 2020). Therefore, in the current study a cut-off value of <0.5 mV was also defined as LA-LVS using multi-electrode mapping catheters with 1 mm electrode size. Furthermore, relevant ACM was defined as a LA-LVS extent of ≥2 cm^2^ in the current study as this cut-off was demonstrated as critical for an increased arrhythmia recurrence rate after PVI in previous studies by our group (Jadidi et al., 2018; Muller-Edenborn et al., 2020). According to these findings, we also used this prespecified cut-off value for the diagnosis of a relevant ACM in the current study. The clinical value of this cut-off was underlined by the fact that patients with an ACM (LA-LVS extent ≥2 cm^2^) in the current study also showed a significantly higher arrhythmia recurrence rate. In contrast, persistent AF patients without ACM (LA-LVS <2 cm^2^) had a high success rate with sinus rhythm maintenance of 80% at 12-months follow-up after PVI.

However, the main limitation of voltage mapping is its invasive nature. Therefore, it can only be applied in patients undergoing LA ablation procedures and is not suited for screening of ACM.

As a non-invasive mapping method for ACM diagnosis Gadolinium-enhanced MRI has been introduced over the last years and some studies reported a correlation of enhanced areas with endocardial low-voltage values (Oakes et al., 2009; Khurram et al., 2014; Marrouche et al., 2014). However, limited availability, costs and difficulties with reproducibility in different centers are significant limitations which were raised by recent studies (Sramko et al., 2015; Chen et al., 2019; Caixal et al., 2021). Therefore, the largest evidence currently exists for endocardial voltage mapping for identification of LA arrhythmogenic tissue.

### Left Atrial Function in Atrial Cardiomyopathy

The atria contribute to cardiac function in various ways: besides the obvious impact of atrial contractility on ventricular filling, the atria exert a reservoir function in times of cardiac overload, are host to important parts of the conduction system and contribute to volume homeostasis through secretion of natriuretic peptides (Goette et al., 2017). Several, individual studies demonstrated that progressive replacement of atrial myocardium with fibrofatty tissue as demonstrated in ACM can affect any of the atrial functions: ACM occurs concomitantly with atrial hypertension as a result of decreased atrial compliance in patients with heart failure (Park et al., 2014; Roh et al., 2020). ACM may also develop secondary to elevated filling pressures (atrial pressure and/or volume overload) in patients with left ventricular/valvular cardiomyopathy. Also, ACM was previously linked to electrical disturbances by progressive intra-atrial conduction (Jadidi et al., 2018; Muller-Edenborn et al., 2020) as well as mechanical dysfunction, with decreased compliance and contractility (Pilichowska-Paszkiet et al., 2018; Seewoster et al., 2019). However, from the previously available data it remains unclear whether these effects occur in parallel or individually.

In healthy individuals, the mean atrial pressures ranged between 2 and 12 mmHg (average 8 mmHg) and normal average v-wave has been determined with 13 mmHg at rest (Braunwald et al., 1961). A maximum p-wave duration of 130 ms as measured on standard 12-lead ECG has been defined as normal (Caceres and Kelser, 1959) and normal strain values are 39% for LASr, 23% for LAScd, and 17% for LASct (Pathan et al., 2017). In the current study, we demonstrate that patients with advanced ACM have a dysfunctional left atrium by several aspects, all being present at the same time: impaired LAP homeostasis with atrial hypertension, electrical conduction slowing and mechanical dysfunction with both reduced atrial compliance/elasticity and impaired passive (during ventricular systole) and active atrial contractile function. These three aspects represent the pathophysiological triad of AF-associated ACM. By application of the calculated pathological cut-off values for the above mentioned left atrial dysfunction we could accurately differentiate between patients with and without relevant ACM underlining the pathophysiological importance of these parameters. Only LAP did not reach significance which might be explained by the fact that patients were under general anesthesia during PVI and volume status might have differed between patients.

### Atrial Cardiomyopathy, Atrial Fibrillation, and Heart Failure

**FIGURE 6:**
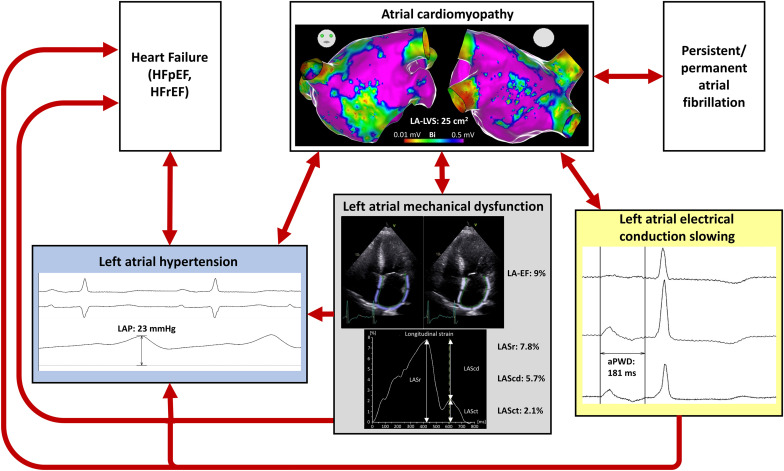
Pathophysiology of atrial cardiomyopathy (ACM). This illustration summarizes the pathophysiology of ACM and the diagnostic possibilities to detect the different pathophysiological mechanisms. The representative patient illustrated has a relevant ACM with a left atrial low-voltage substrate (LA-LVS) at 0.5 mV threshold of 25 cm^2^, a hypertensive left atrial pressure (LAP) of 23 mmHg, an impaired left atrial emptying fraction (LA-EF) of 9%, an impaired left atrial strain during reservoir phase (LASr) of 7.8%, during conduit phase (LAScd) of 5.7%, and during contraction phase (LASct) of 2.1% as well as a prolonged amplified p-wave duration (aPWD) of 181 ms.

The findings in the current study supports the clinical observation that AF, once it occurs for the first time, often perpetuates itself, leading from initially paroxysmal phenotypes to persistent and permanent forms (“AF begets AF”) (Wijffels et al., 1995; Allessie, 1998). As we and others found ACM in a significant part of persistent AF-patients (Platonov et al., 2011; Haissaguerre et al., 2016), it seems reasonable that a vicious circle of left atrial hypertension, associated or due to mechanical dysfunction of the LA, promotes progressive electrical atrial disease and finally progression to permanent AF (Figure 6). AF itself thereby leads to remodeling, causing electrical, contractile, and structural changes which complicates an effective AF elimination in progressive stages (Wijffels et al., 1995; Everett et al., 2006). Numerous clinical trials demonstrate that patients with ACM have higher recurrence rates following AF-ablation (Verma et al., 2005; Oakes et al., 2009; Rolf et al., 2014; Jadidi et al., 2016). Our current study cohort confirms these findings, and arrhythmia recurrence was 3.8-fold higher in patients with ACM as evidenced using endocardial voltage mapping. Any of the abovementioned atrial function parameters show a clear trend to identify patients with particularly high or low risk for recurrences. An IAAT ≥143 ms as marker for electrical conduction slowing and a LA-EF ≤33 and LASct ≤6.8% as markers for mechanical dysfunction were significant predictors of arrhythmia recurrence after PVI.

In line with previous studies, the current study shows that the non-invasive parameters LA-EF, LAS, and aPWD might enable an early diagnosis of ACM and thus allow a timely identification and treatment of patients at risk for new-onset AF (Jadidi et al., 2018; Pilichowska-Paszkiet et al., 2018; Seewoster et al., 2019; Muller-Edenborn et al., 2020). The current findings might also serve as an explanation for the fact that reduced LASr and prolonged aPWD were reliable markers to predict new-onset AF in patients with heart failure with preserved ejection fraction (HFpEF) (Muller-Edenborn et al., 2020; Park et al., 2020) or could be detected more frequently in patients with persistent AF than in those with paroxysmal AF or those without history of AF (Reddy et al., 2020).

In addition, ACM might be important in patients with stroke (King et al., 2017; Muller et al., 2018). This might also explain that a very recent study by Liao et al. (2020) demonstrated that reduced LASr (measured during AF) enabled identification of patients at risk for future ischemic stroke (Liao et al., 2020). This underlines the importance of valid non-invasive screening methods in order to identify patients at risk as early as possible and to prevent ACM-associated complications.

### Limitations

First, the current study is performed in patients with known AF, and it remains unclear whether these observations also apply for ACM in patients without AF. Second, arrhythmia recurrence was assessed by 72-hour Holter ECG 6 and 12 months after PVI only. Therefore, arrhythmia recurrence might be underestimated in the current approach. Nevertheless, in case of symptoms an event recorder was used additionally. Third, voltage maps were acquired using either a 20-polar Lasso-Nav or a PentaRay-Nav mapping catheter for high-density voltage mapping in the current study. New multi-electrode catheters (e.g., Advisor HD Grid and the INTELLAMAP ORION) might reduce influence of wave front orientations on variability in bipolar electrogram characteristics. Nevertheless, the current study is in concordance with previous studies underlining the clinical value of ACM-diagnosis with a low-voltage cut-off <0.5 mV based on high-density voltage mapping in sinus rhythm using the above-mentioned mapping catheters. Fourth, due to the small sample size included in the current study we did not subdivide the patient cohort into different ACM stages. Future large-scale studies are needed to provide data on LAP, left atrial electrical and mechanical function depending on different ACM stages.

## Conclusion

Left atrial hypertension, electrical conduction slowing and mechanical dysfunction are associated with ACM. The current findings improve the understanding of ACM pathophysiology and might allow the identification of patients at risk for new-onset AF and the development of appropriate therapeutic strategies to prevent AF and its associated complications.

## Data Availability Statement

The original contributions presented in the study are included in the article/Supplementary Material, further inquiries can be directed to the corresponding author/s.

## Ethics Statement

The studies involving human participants were reviewed and approved by the Ethics Committee at the University of Freiburg. The patients/participants provided their written informed consent to participate in this study.

## Author Contributions

ME, BM-E, DT, TA, and AJ contributed to conception and design of the study. ME, BM-E, NJ, MA, HL, JA, TA, and AJ collected the data. ME, BM-E, and AJ performed the statistical analysis. ME, BM-E, and AJ wrote the first draft of the manuscript. All authors contributed to manuscript revision and, read and approved the submitted version.

## Conflict of Interest

The authors declare that the research was conducted in the absence of any commercial or financial relationships that could be construed as a potential conflict of interest.

## Publisher’s Note

All claims expressed in this article are solely those of the authors and do not necessarily represent those of their affiliated organizations, or those of the publisher, the editors and the reviewers. Any product that may be evaluated in this article, or claim that may be made by its manufacturer, is not guaranteed or endorsed by the publisher.
