# Supplementary material for: Left Atrial Hypertension, Electrical Conduction Slowing, and Mechanical Dysfunction – The Pathophysiological Triad in Atrial Fibrillation-Associated Atrial Cardiomyopathy
Source: Front Physiol. 2021 Aug 5;12:670527. doi: 10.3389/fphys.2021.670527 (PMC8375593; doi:10.3389/fphys.2021.670527)
Supplement: Supplementary file 1 [file Image_1.pdf]

Supplementary Material

1 Supplementary Figures and Tables

1.1 Supplementary Figures

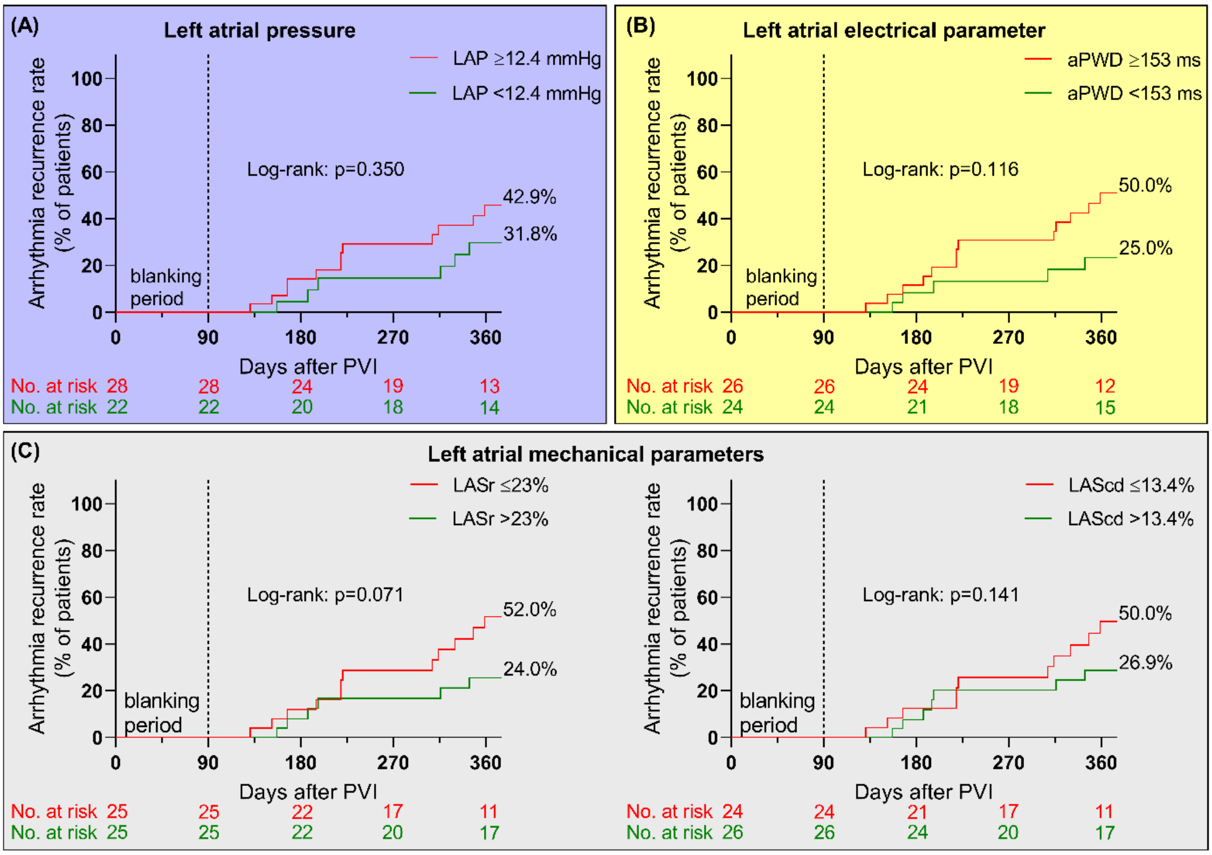

Supplementary Figure 1. Kaplan-Meier curves illustrate arrhythmia recurrence after pulmonary vein isolation (PVI) in patients with left atrial hypertension (left atrial pressure [LAP] ≥12.4 mmHg, red curve in Panel (A)), prolonged left atrial electrical parameters (amplified p-wave duration [aPWD] ≥153 ms, red curve in Panel (B)) and impaired left atrial mechanical parameters (left atrial strain during reservoir phase [LASr] ≤23% and left atrial strain during conduit phase [LAScd] ≤13.4%, red curves in Panel (C)) compared to patients with normal cut-offs (green curves).
